# Application of kefir-isolated *Lactiplantibacillus plantarum* and *Lacticaseibacillus paracasei* to obtain a cashew-based fermented beverage with enhanced anti-inflammatory properties

**DOI:** 10.3389/fmicb.2026.1763414

**Published:** 2026-03-10

**Authors:** Luz Pietrantuono, Ana Clara Sabbione, Carolina Dardis, Graciela L. Garrote, Ana Agustina Bengoa

**Affiliations:** 1 Centro de Investigación y Desarrollo en Ciencia y Tecnología de Alimentos (CIDCA, CONICET-CICPBA-UNLP), La Plata, Argentina; 2 Facultad de Ciencias Exactas, Universidad Nacional de La Plata, La Plata, Buenos Aires, Argentina

**Keywords:** anti-inflammatory properties, cashew nut, fermented beverage, kefir, milk analog, plant-based food, probiotics

## Abstract

The increasing demand for healthy non-conventional probiotic foods has positioned plant-based milk analogs as viable alternatives to traditional dairy products. In these matrices, fermentation with probiotic lactic acid bacteria (LAB) offers the opportunity to develop new functional foods with enhanced sensory, nutritional, and health properties. The present study aimed to develop a functional probiotic beverage by fermenting a cashew-based matrix with LAB isolated from kefir grains, characterizing its techno-functional and anti-inflammatory properties. A cashew-based beverage was prepared and subjected to various heat treatments before fermentation assays. *Lacticaseibacillus paracasei* CIDCA 8339 and CIDCA 83124, *Lactiplantibacillus plantarum* CIDCA 8327, and *Lactococcus lactis* subsp. *lactis* CIDCA 8221 were evaluated for beverage fermentation at 30°C. All strains achieved high counts (8–9 log CFU/mL), but only CIDCA 8327 and CIDCA 8339 reduced the pH to approximately 5.00–5.20. Notably, *L. plantarum* CIDCA 8327 demonstrated the ability to inhibit the growth of undesirable spore-forming microorganisms that survive pasteurization. The mixed-culture of CIDCA 8327 and CIDCA 8339 enhanced LAB growth and acidification (pH 4.53 after 24 h). This effect occurred with increased lactic and acetic acid production. This resulting fermented beverage exhibited a notably high protein (4.7%), fiber (1.8%), and lipid content (7.8%, with over 75% of unsaturated fatty acids) compared to commercial plant-based fermented beverages. The product exhibited colloidal stability for at least 1 month during refrigeration and showed a higher apparent viscosity (130.5 mPa.s) than its unfermented counterpart (102 mPa.s). Additionally, the non-microbial fraction from the fermented beverage successfully suppressed the inflammatory response by 85% in a Caco-2-ccl20:luc reporter system, a significant improvement over the 50% reduction seen with the unfermented product. This suggests that LAB produce bioactive metabolites, such as organic acids, which enhance the immunomodulatory effects. In conclusion, the cashew-based matrix is highly suitable for kefir-isolated LAB. However, selecting an appropriate starter culture is crucial for achieving a product with a low spoilage microbial load. In this context, fermentation with the mixed starter CIDCA 8327 and CIDCA 8339 resulted in a nutritious probiotic beverage with enhanced techno-functional characteristics and anti-inflammatory properties, indicating its potential as a functional food to support gastrointestinal health.

## 1 Introduction

The field of functional foods has garnered consumers’ attention in recent years, emphasizing the role of diet in maintaining a healthy state and increasing interest in probiotic-containing foods. Probiotics are defined as “live microorganisms which when administered in adequate amounts confer a health benefit on the host” (Hill et al., 2014). Health-promoting properties associated with probiotics include inhibition of intestinal pathogens, antioxidant activity, reduction of serum cholesterol, and modulation of the immune response (Bengoa et al., 2021). Dairy products, such as yogurt, cheese, and fermented milks, are the most common carriers for probiotic microorganisms available in the market. However, nowadays, a rising consumer demand has promoted the search and development of new non-dairy probiotic foods, particularly for people who suffer from lactose intolerance, milk protein allergies, or hypercholesterolemia. Additionally, the need for these alternative probiotic products is driven by the growing population preference for a whole plant-based diet, motivated by health implications, animal welfare issues, and environmental concerns (De Bellis et al., 2021; Hidalgo-Fuentes et al., 2024).

Among the diverse segments of the plant-based market, the category of milk analogs shows one of the most significant growth projections. Plant-based beverages are water extracts produced from legumes, nuts, seeds, cereals, or pseudocereals (Lavelle et al., 2025). Among the wide range of raw materials that can be used, cashew nuts stand out due to their excellent nutritional qualities. They are rich in macronutrients, containing significant amounts of fat, carbohydrates, protein, and total dietary fiber. In terms of their lipid fraction, cashew nuts are rich in mono and polyunsaturated fatty acids with a high content of oleic and linoleic acids (Rebouças et al., 2016; Nogueira Oliveira et al., 2019). Furthermore, cashew nuts provide essential amino acids for adults and children, as well as micronutrients like iron, phosphorus, magnesium, potassium, vitamins (E and K), and bioactive compounds such as phenolics and flavonoids (Rebouças et al., 2016; Dantas and Pontes Costa, 2022). Regular consumption of cashew nuts has been linked to diverse benefits for human health, including lowering blood cholesterol levels, controlling diabetes and coronary heart disease risk, maintaining healthy bones, and preventing high blood pressure (Shori and Al Zahrani, 2022).

The global market of plant-based beverages is currently undergoing a significant expansion with an expected annual growth rate of 15% from 2023 to 2028 (Hidalgo-Fuentes et al., 2024). By 2020, retail sales had reached approximately $2.4 billion, with a projected growth to $62 billion by 2030 (Mekanna et al., 2024). In Argentina, the consumption volume of plant-based beverages was recorded at 3.5 million liters in 2021, and it is projected to increase fourfold, reaching 14 million liters by 2026 (Alimentos Argentinos, 2024). However, factors such as inadequate nutritional quality, unpleasant aromas and flavors, and poor texture of these products often limit consumers’ acceptance (Tangyu et al., 2019). In this context, fermentation emerges as a promising strategy for developing novel beverages with improved characteristics.

Lactic acid bacteria (LAB) have been utilized for thousands of years to prolong the shelf life of products through fermentation. These bacteria convert sugars present in the matrix into lactic acid that imparts sensory properties and contributes to product preservation. As a result, fermented foods have a low risk of microbial contamination. Fermentation also improves the nutritional and organoleptic properties of these beverages, providing flavors and textures that are completely different from those present in the starting materials (Terpou et al., 2025). The process can enhance nutrient digestibility and availability, primarily due to the proteolytic activity of the microorganisms and the biosynthesis of vitamins. Moreover, it helps to reduce anti-nutritional factors, such as phytic acid and saponins, which further improves nutrient bioavailability. In addition, fermentation can improve the beverage taste and aroma through the production of flavor compounds, including organic acids and volatile metabolites, while simultaneously decreasing off-flavor components usually present in these food matrices (Tangyu et al., 2019; Hidalgo-Fuentes et al., 2024).

The fermentation process of plant-based beverages can differ based on the specific matrix used. Therefore, selecting the proper starter for each matrix is crucial for producing high-quality products. LAB are excellent candidates for this purpose due to their versatility in adapting to different matrices, their safe and traditional use in food fermentation, and their potential probiotic properties (Montemurro et al., 2021). Kefir is a homemade fermented beverage with a long tradition of consumption and numerous health benefits. It is produced by fermenting milk with kefir grains, which are composed of a complex community of LAB, acetic acid bacteria, and yeasts associated with a matrix of proteins and polysaccharides. These kefir grains serve as a natural reservoir of safe and potentially probiotic strains that can be isolated and characterized for their application in the development of novel functional products with validated health claims (Bengoa et al., 2019b). The growing demand for healthy foods drives continuous innovation in non-conventional probiotic foods. Therefore, fermenting plant-based beverages with probiotic LAB isolated from kefir presents the opportunity to create new functional foods with enhanced sensory qualities, nutritional properties, and health benefits. In this context, the present work aimed to evaluate the potential application of probiotic LAB isolated from kefir grains to obtain a cashew nut-based fermented beverage and characterize the techno-functional and *in vitro* anti-inflammatory properties of the final product.

## 2 Materials and methods

### 2.1 Bacterial strains and culture conditions

All LAB strains used in the present study were isolated from kefir grains and belong to the Centro de Investigación y Desarrollo en Ciencia y Tecnología de Alimentos (CIDCA) collection. *Lactiplantibacillus plantarum* CIDCA 8327, *Lacticaseibacillus* paracasei CIDCA 8339 and CIDCA 83124, and *Lactococcus lactis* subsp. *lactis* CIDCA 8221 were stored at −80°C in sterile skim milk at 50% v/v (La Serenísima, Mastellone Hnos S.A, General Rodriguez, Argentina). Lactobacilli strains were grown in MRS broth while TYL broth (Tryptone 10 g/L, Yeast extract 10 g/L and Lactose 10 g/L) was used for lactococci. All strains were incubated for 24 h at 30°C under aerobic conditions.

### 2.2 Cashew nut beverages

#### 2.2.1 Preparation of cashew nut beverage

Cashew nuts (100 g) were sterilized with distilled water (300 mL) in an autoclave at 121°C for 15 min. The soaking water was removed, and the nuts were rinsed with sterile water. Sterile water (300 mL) was then added, and the mixture was blended for 3 min (Turboblender TB 79, Argentina). The slurry was filtered through a sterile cloth. The final volume was 400 mL. The beverage was divided into aliquots and subjected to different heat treatments: 121°C for 15 min, 100°C for 30 min, or two successive treatments at 80°C for 30 min. The treatment that yielded a beverage with low microbial load, good appearance, and adequate fluidity was selected for further studies.

#### 2.2.2 Fermentation of cashew nut beverages

Lactic acid bacteria fresh cultures (24 h at 30°C) were inoculated into the cashew-based beverage (CBB) as single-cultures or mixed-cultures at an initial concentration of approximately 1.0 × 107 CFU/mL. Fermentations were conducted in the CBB, either without added sugar or supplemented with 1% sucrose, and incubated at 30°C for 24 or 48 h.

The fermented products were characterized based on pH, organoleptic attributes, and microbial counts to select the beverage that exhibited the most favorable properties for further study. Organoleptic characteristics as color, texture, and aroma of each fermented beverage, were evaluated. The pH was measured using a pH meter with an electrode (Mettler Toledo, United States) after 24 and 48 h of incubation. LAB count was performed before and after fermentation; serial 1:10 dilutions were prepared in physiological solution and CFU were determined using the drop-plate method. Moreover, total mesophilic counts were determined on nutrient agar using the drop-plate method. Colonies grown in nutrient agar at 30°C were isolated and Gram stained.

### 2.3 Molecular identification of spore-forming bacterial isolate

Genomic DNA was extracted using the QIAamp PowerFecal Pro DNA Kit (Qiagen, Hilden, Germany) from overnight bacterial culture grown in nutrient broth. DNA concentration and purity were assessed with a NanoDrop 2000 spectrophotometer (Thermo Scientific, United States) by measuring A260, A260/280, and A260/230 ratios prior to PCR amplification.

The 16S rRNA gene was amplified using universal primers 27F (5’-AGAGTTTGATCCTGGCTCAG-3’) and 1492R (5’-GGTTACCTTGTTACGACTT-3’). PCR reactions were performed in a total volume of 25 μL containing 1X buffer supplied with the enzyme (100 mM Tris-HCl, 500 mM KCl, pH 9), 0.5 μM of each primer, 2.5 mM MgCl_2_, 0.2 mM dNTPs, 0.05 U/μL Taq DNA polymerase (Inbio Highway, Tandil, Argentina), and 0.5 ng/μL template DNA. Amplifications were carried out in a MyCycler Thermal Cycler (Bio-Rad, Hercules, CA, United States). The PCR program consisted of an initial denaturation at 94°C for 5 min, followed by 35 cycles of denaturation at 92°C for 30 s, annealing at 58°C for 45 s, and extension at 72°C for 1.5 min, with a final extension at 72°C for 4 min.

PCR products were analyzed by electrophoresis on 1% (w/v) agarose gels prepared in TAE buffer (4.84 g/l Tris-base, 5.71% v/v glacial acetic acid, 0.05 M EDTA, pH 8.0), stained with ethidium bromide, and visualized under UV light (LABNET TM-26). Fragment size was estimated using a DNA Ladder Mix (New England Biolabs). The ∼1,500 bp band was excised and purified for sequencing at Macrogen (South Korea) using the same amplification primers. The obtained sequences were assembled into a contig using Vector NTI, and the resulting consensus sequence was identified using BLASTn.^1^ The gene sequence was deposited in Genbank (NCBI, Accession number PX987639). A phylogenetic analysis based on 16S rRNA gene sequences was performed in MEGA X (v11) using the Neighbor-Joining method and Kimura two-parameter distances. Bootstrap support was estimated with 1,000 replicates, and a bootstrap consensus tree is shown. *Bacillus cereus* was included as the outgroup.

### 2.4 Organic acid analysis

Organic acids in the fermented beverages were qualitatively and quantitatively determined by ion exchange high-performance liquid chromatography using an AMINEX HPX-87H ion exchange column (BioRad Labs, Richmond, CA 94804, United States) coupled to an UV detector at 214 nm (Waters 996, Millipore Corporation, Milford, MA 01757, United States). For sample preparation, 1 mL of product was first centrifuged for 20 min at 4,000 × g and 4°C, and then for 30 min at 10,000 × g and 4°C. The supernatant was filtered through a 0.45 μm membrane (Millipore Corporation, United States), diluted, and injected into the chromatograph. The analysis was performed at a flow rate of 0.7 mL/min and 60°C using 0.009 N H_2_SO_4_ as the mobile phase. Acid standards (Sigma-Aldrich, St. Louis, MO, United States) were used for the identification and quantification of organic acids. All measurements were performed at least in triplicate.

### 2.5 Cashew-based beverage and cashew fermented beverage characterization

#### 2.5.1 Centesimal composition

Ash and moisture content were determined by gravimetric AOAC methods (AOAC, 1990). Total protein determination of the beverages was performed using the Kjeldahl method (conversion factor was 5.5 g protein/g nitrogen). Total lipids were determined based on the Soxhlet extraction method with petroleum ether (AOAC, 1990). Total dietary fiber was analyzed with a Megazyme K-TDFR-200A enzymatic kit, method 991.43 (AOAC, 1991), and carbohydrate content was determined by difference [100% - (%moisture + % ash + % protein + % lipids + % total dietary fiber)]. Energy content was calculated according to Atwater factors, with 4 kcal/g carbohydrates, 4 kcal/g protein, and 9 kcal/g lipids.

#### 2.5.2 Fatty acid profile

Samples (1 g) were weighed and mixed with 10 mL of Folch solution (chloroform–methanol, 2:1, v/v). After 12 h, the samples were filtered, and 1.2 mL of distilled water was added. The upper phase was discarded. The lower chloroform phase containing total lipids was dried under a nitrogen stream. HCl/methanol containing sodium sulfate (19:1, v/v; 4 mL) was then added, and the samples were heated at 80°C for 10 min. After cooling, hexane (1 mL) was added. The mixture was extracted and transferred to vials for gas chromatography analysis.

Fatty acid methyl esters (FAMEs) were analyzed using an Agilent Technologies 7,890 A gas chromatograph equipped with a DB-23 capillary column (30 m length, 0.25 mm i.d., 250 μm film thickness). Injector and detector temperatures were set at 250 and 280°C, respectively. The temperature program was as follows: initial temperature 50°C (1 min), ramped at 25°C/min to 175°C, then at 4°C/min to 230°C, held for 15 min. Total run time: 34.75 min. FAMEs were identified using an external standard (Supelco 37 Component FAME Mix, 100 mg neat, Cat. No. 18919-1AMP).

#### 2.5.3 Technofunctional properties

##### 2.5.3.1 Rotational viscometry

Measurements were performed using a Discovery HR-20 hybrid rheometer (TA Instruments, New Castle, United States) with a plate–plate geometry. An aliquot of the product was placed on the lower plate, and the upper plate was lowered to a preset gap of 1 mm. The sample was thermostatted at 25°C and subjected to a flow cycle consisting of an acceleration of 167 s ^–2^ to reach a shear rate of 500s^−1^ in 2 min. This shear rate was maintained for 1 min, then decreased to 0 s ^−1^ using the same acceleration in the negative direction. Shear stress was recorded as a function of shear rate, and apparent viscosity (η_ap_) was determined at a shear rate of 300 s ^−1^ from the corresponding flow curves. All measurements were performed at least in duplicate.

##### 2.5.3.2 Colloidal stability

Colloidal stability was assessed by kinetic destabilization using light scattering through a vertical scan optical analyzer (Beckman Coulter, United States). For time-dependent stability analysis, samples were loaded into cylindrical glass cells, and backscattering profiles (%BS) were monitored immediately along the sample height (approximately 60 mm). Samples were then stored at 4°C for approximately 30 days, and measurements were taken every 3 days. All samples were analyzed in duplicate.

##### 2.5.3.3 Superficial color

The superficial color was evaluated using a surface colorimeter (Minolta Optics Inc. CR Series 300, Japan). Ten color measurements were taken directly on the beverage surface and the CIE Lab* color space (CIE, Commission Internationale de l’Éclairage) was applied, recording lightness (L), ranging from 0 (black) to 100 (white), and chromatic coordinates a* (red–green) and b* (yellow–blue) (Macdougall, 2010).

Hue and ΔE were calculated using the Equations 1, 2, respectively:


$$H\,U\,E=\frac{A\,r\,c\,t\,g\,\frac{b*}{a*}*180}{3.14}+180$$ (1)

ΔE=[(L-1L)2+2(a-1a)2+2(b-1b)2]21/2 (2)

### 2.6 Modulation of intestinal inflammatory response *in vitro*

#### 2.6.1 Preparation of non-microbial fraction of cashew fermented beverage

To obtain the non-microbial fractions, samples were centrifuged for 20 min at 4,000 g and 4°C (Hermle Z 326 K centrifuge). A control was also prepared by acidifying CBB to pH 4.6 with lactic acid (50 mM). The resulting supernatants were centrifuged twice at 10,000 g and once at 7,000 g for 30 min at 4°C, neutralized, sterilized by filtration (0.45 μm pore membrane, Millipore Corporation, United States), and diluted 1:1 with DMEM.

#### 2.6.2 Modulation of the intestinal innate immune response in Caco-2 ccl20:luc cells assay

The caco-ccl20:luc reporter system consists of Caco-2 intestinal epithelial cells stably transfected with a luciferase reporter construction under the control of the chemokine-ligand-20 (CCL20) promoter (Romanin et al., 2010).

Non-microbial fraction of cashew fermented beverage (CFB) and CBB (200 μL) was added to wells containing confluent Caco-2 ccl20:luc cells. Plates were incubated for 30 min at 37°C in a 5% CO_2_–95% air atmosphere. *Salmonella enterica serovar Enteritidis* flagellin (1 μg/mL) was then added to the appropriate wells, followed by a 5 h incubation under the same conditions. Controls included: (i) DMEM diluted 1:1 with PBS as the untreated basal control, and (ii) a positive control with flagellin to define 100% inflammatory stimulation. After incubation cells were lysed with a lysis buffer (Promega, United States). Luciferase activity was measured in a Labsystems Luminoskan TL Plus luminometer (Thermo Scientific, United States) using a luciferase assay system (Promega, Madison WI, United States). All measurements were performed in triplicate. Luminescence values were normalized to the stimulation control and expressed as a percentage of the normalized average luminescence (% NAL) ± standard deviation (SD).

### 2.7 Statistical analysis

Variance analysis (ANOVA) was performed using GraphPad Prism 8. Multiple comparisons were conducted using Tukey’s test and *t*-test with a significance level of 0.05.

## 3 Results

### 3.1 Cashew nut beverage

**FIGURE 1:**
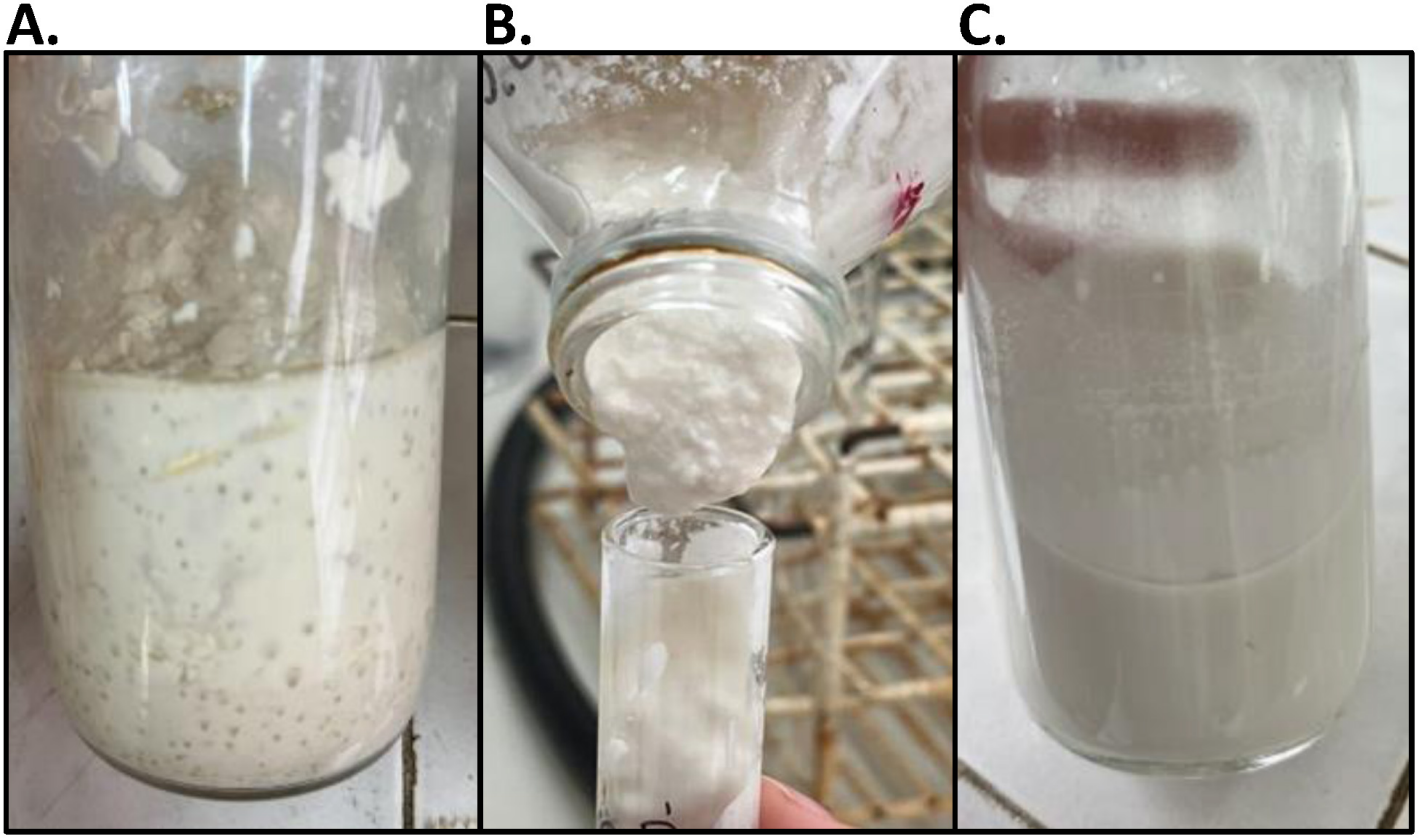
Cashew-based beverage (CBB) after different heat treatments: 121°C for 15 min **(A)**, 100°C for 30 min **(B)**, and two successive treatments at 80°C for 30 min **(C)**.

Cashew nut beverage was prepared and subjected to different heat treatments to select the most appropriate for this matrix. It was evident that heat treatments above 100°C produced changes in texture, probably due to protein denaturation. As a result, the product was thick and non-fluid. In contrast, two successive treatments at 80°C resulted in a fluid beverage (Figure 1). Moreover, the latter treatment showed a microorganisms’ load below the detection limit (<10 CFU/mL), which guarantees the safety and prolonged shelf life of the product.

### 3.2 Selection of starter culture for cashew nut beverage fermentation

**FIGURE 2:**
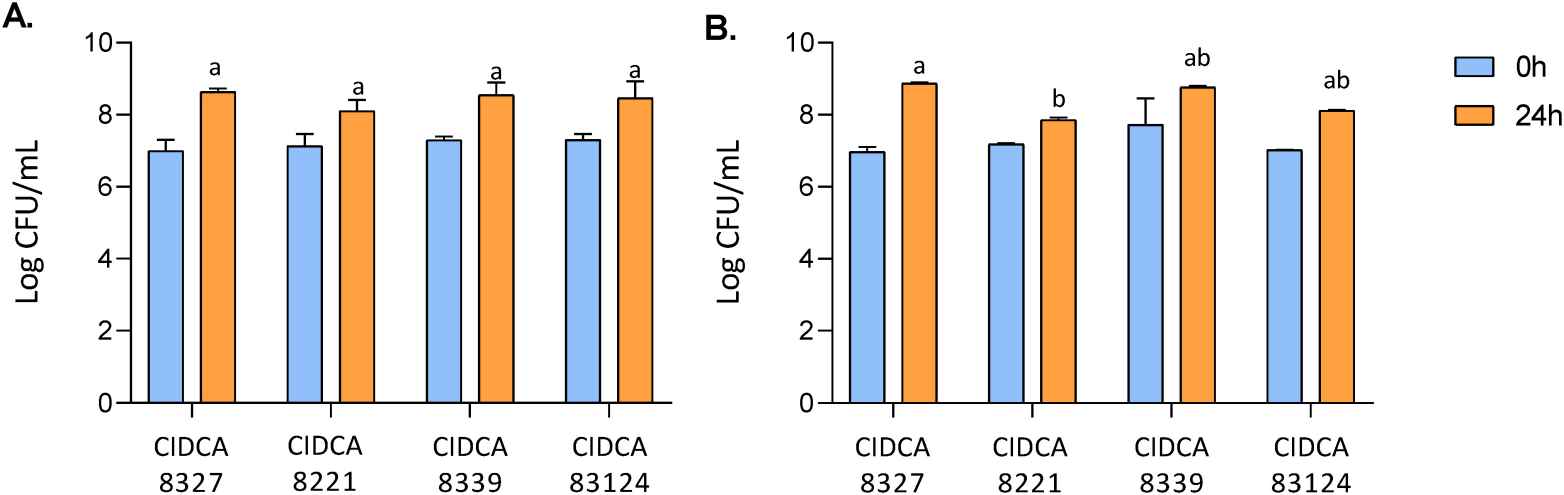
LAB grown in cashew-based beverage (CBB) **(A)** and CBB 1% sucrose **(B)** after 24 h incubation at 30°C. Different letters indicate significant differences (*p* < 0.05).

Four LAB strains isolated from kefir grains were evaluated as starters. Fermentation was tested in the CBB, and the CBB added with sucrose 1%. Sucrose was added due to the low levels of fermentable sugars in nut-based beverages, which may limit LAB growth and acidification (Huang et al., 2023; Lima Araujo Filho et al., 2023). Figures 2A,B show the initial and final log CFU/mL values for each strain in the cashew beverage and in the sucrose-supplemented beverage, respectively. All the LAB tested were able to grow in the beverage, reaching between 8 and 9 log CFU/mL, without showing significant differences with the addition of sucrose in any case (*p* > 0.05). In the beverage without sucrose, the growth of all four strains was similar. However, when sugar was added, *Lactococcus lactis* CIDCA 8221 showed a slightly lower final count than *Lactiplantibacillus plantarum* CIDCA 8327 (Figure 2B). Regarding pH values, no substantial pH reduction was detected with *Lactococcus lactis* CIDCA 8221 and *Lacticaseibacillus paracasei* CIDCA 83124, even after 48 h fermentation, whether sucrose was added or not (Table 1). *L. plantarum* CIDCA 8327 showed the lowest pH values at 24 h, with a marked reduction under both conditions. In contrast, *L. paracasei* CIDCA 8339 exhibited greater acidification in the presence of sucrose.

**TABLE 1:** Total mesophilic microorganisms (log CFU/mL) and pH of the heat treated cashew-based beverage (CBB) fermented with different LAB strains.

	Cashew based beverage			Cashew based beverage with 1% sucrose		
Lactic acid bacteria	Total mesophilic microorganism (log CFU/mL)	pH		Total mesophilic microorganisms (log CFU/mL)	pH	
		24 h	48 h		24 h	48 h
CIDCA 8327	ND	5.17	5.02	ND	4.65	4.79
CIDCA 8221	ND	5.96	5.68	ND	5.97	5.45
CIDCA 8339	5.50 ± 0.10 ^b^	5.48	5.21	5.41 ± 0,02 ^b^	4.41	4.22
CIDCA 83124	5.22 ± 0.06 ^b^	5.90	5.66	5.60 ± 0,10 ^b^	5.90	5.62
Control*	7.23 ± 0.02 ^a^	6.33	6.38		−	−

*Control corresponds to cashew-based beverage incubated at 30 °C for 24–48 h without any starter added. ND, not detected (Detection limit = 500 CFU/mL). Different letters indicate significant differences (Tukey’s test *p* < 0.05).

The heat-treated cashew beverage without added starter was incubated at 30°C for 24 h to assess the growth of heat-resistant microorganisms. After incubation, pH and total mesophilic counts on nutrient agar were determined (Table 1). Although pH did not change, incubation led to significant microbial growth, reaching 7.23 log CFU/mL. However, the appearance and aroma of the beverage remained unchanged. Gram staining revealed that the contaminating microorganisms were spore-forming Gram-negative bacilli. The spore-forming Gram-negative bacillus was isolated and identified as *Paenibacillus* based on 16S rRNA gene sequencing using Blastn. Phylogenetic analysis grouped the *Paenibacillus* isolated strain with *Paenibacillus xylanivorans* (Supplementary Figure 1). Nonetheless, 16S rRNA analysis alone is insufficient to resolve the species with confidence. When the beverage was fermented with LAB, *Paenibacillus* growth was partially or completely inhibited. Table 1 shows that fermentation with *L. paracasei* CIDCA 8339 and CIDCA 83124 resulted in a reduction of 2 log CFU/mL in the total mesophilic count compared to the control. Furthermore, fermentation with *L. plantarum* CIDCA 8327 or *Lc. lactis* CIDCA 8221 reduced counts on nutrient agar below the detection limit. This result suggests competition for nutrients or the production of antimicrobial compounds that inhibit spore-forming bacilli in the matrix.

**FIGURE 3:**
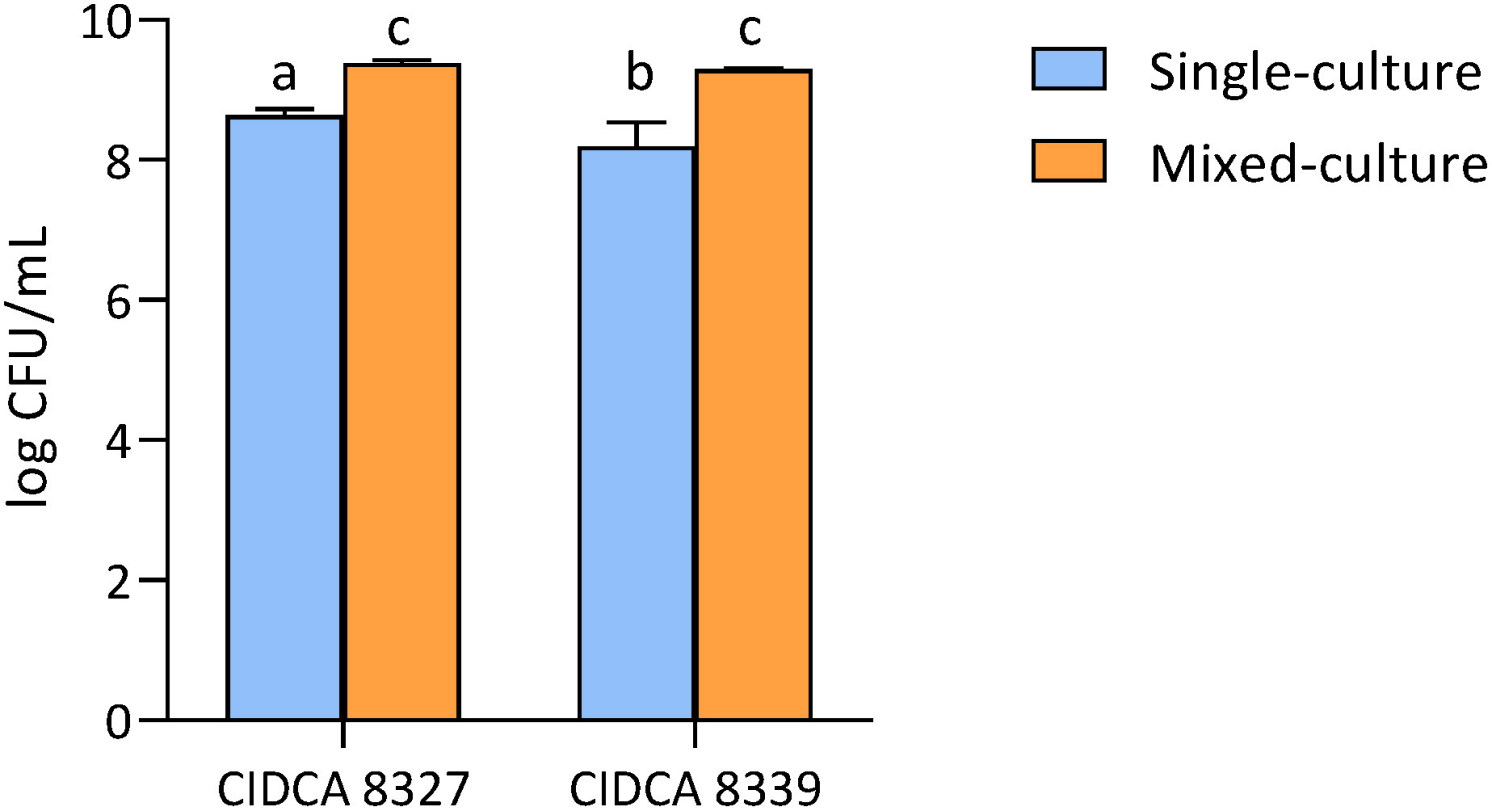
CIDCA 8327 and CIDCA 8339 final concentration in cashew-based beverage (CBB) fermented with single-culture or mixed-culture. Initial concentration was normalized at 7 log CFU/mL. Different letters indicate statistical differences among the data (*p* < 0.05; Tukey’s multiple comparison test).

Based on these results, *L. plantarum* CIDCA 8327 was selected for fermentation due to its inhibitory effect on spore-forming microorganisms and its ability to grow and acidify the product. *L. paracasei* CIDCA 8339 also showed good fermentation performance and produced a desirable yogurt-like aroma. Therefore, fermentation using a mixed-culture of CIDCA 8327 and CIDCA 8339 as the starter was evaluated. These beverages were fermented without added sucrose, as its inclusion did not significantly improve fermentation. Omitting sucrose also simplified production by removing one processing step. In addition, consumers perceive beverages without added sugar as healthier than those labeled as containing added sugar (Prada et al., 2021). The mixed-culture significantly enhanced the growth of both LAB, reaching higher final concentrations than single cultures (Figure 3). In addition, pH values improved (Table 2). The presence of *L. paracasei* CIDCA 8339 during fermentation helped achieve a pH close to 4.5 within 24 h, a critical threshold for fermented food safety. Moreover, the combined starter produced a beverage with an enhanced aromatic profile compared with fermentation using CIDCA 8327 alone. Organic acid analysis showed that the combined LAB significantly increased lactic acid (52.8 mM) and acetic acid (6.91 mM) levels in the final product. These increases correlated with the lower pH. Butyric acid levels were approximately 6–8 mM in both fermented beverages. This result indicates that *L. plantarum* CIDCA 8327 contributes to butyric acid production when used alone or in a mixed-culture (Table 2). Based on these findings, the mixed-culture of CIDCA 8327 and CIDCA 8339 was selected as a suitable starter for cashew beverage fermentation and further characterization.

**TABLE 2:** pH and organic acid levels of cashew fermented beverage (CFB) with single-culture CIDCA 8327 or the mixed-culture CIDCA 8327 and CIDCA 8339.

Sample	pH	Organic acids (mM)		
		Lactic acid	Acetic acid	Butyric acid
CBB	6.33 ± 0.11^a^	ND	ND	ND
CIDCA 8327	5.16 ± 0.22^b^	21.70 ± 3.19^a^	ND	8.28 ± 1.42
CIDCA 8327 and CIDCA 8339	4.32 ± 0.32^c^	52.80 ± 6.21^b^	6.91 ± 0.35	5.96 ± 1.77

ND, not detected. Different letters in the same column indicate significant differences (pH values: Tukey’s test *p* < 0.05; organic acids: *t*-test *p* < 0.05).

### 3.3 Centesimal composition and fatty acid profile

Table 3 presents the proximate composition of the cashew-based beverage (CBB) and cashew beverage fermented with the mixed-culture (CFB), which was equivalent for both products. Regarding moisture content, CBB and CFB showed values of 81.75 and 81.21%, respectively. These percentages are slightly lower than those of similar plant-based products (Reyes-Jurado et al., 2021), likely due to the higher proportion of cashew nuts used in the preparation of these beverages. Consequently, high percentage of solids was observed. The beverages exhibit 4–5 g/100 mL of protein, 8–9 g/100 mL of lipids, and 2–3 g/100 mL of carbohydrates. Total dietary fiber was also high with a content closed to 2%. Despite the high proportion of lipids present in these beverages, the analysis of the fatty acids profile (Table 4) revealed that over 75% corresponds to unsaturated fatty acids. The beverages exhibit oleic (58–60%), linoleic (16–17%), palmitic (14–15%), and stearic acids (7–8%) as the most abundant fatty acids. Moreover, Table 4 shows a high oleic/linoleic acid ratio in both beverages. This ratio is technically relevant because higher oleic acid levels increase resistance to rancidity and improve shelf life.

**TABLE 3:** Cashew-based beverage (CBB) and cashew fermented beverage (CFB) proximate composition (% w/w) and Calories (Kcal/100 g).

Components	CBB	CFB
Proteins	4.676 ± 0.005 ^a^	4.7 ± 0.1 ^a^
Lipids	9 ± 1 ^a^	7.8 ± 0.9 ^a^
Total dietary fiber	2.1 ± 0.3 ^a^	1.82 ± 0.06 ^a^
Ashes	0.408 ± 0.002 ^a^	0.423 ± 0.004 ^a^
Moisture	81.3 ± 0.4 ^a^	81.2 ± 0.1 ^a^
Carbohydrates*	2.476	4.037
Calories	114	109

*Estimated by difference. Different letters in the same row indicate significant differences (Tukey’s test *p* < 0.05).

**TABLE 4:** Cashew-based beverage (CBB) and cashew fermented beverage (CFB) percentage fatty acid profiles.

Sample	16:0	18:0	18:1 ω -9	18:2 ω -6	18:3 ω -3	20:0	SFA	UFA	O/L
**CBB**	14.4 ± 0.3^a^	7.6 ± 0.6^a^	59.5 ± 1.7^a^	16.9 ± 1.0^a^	ND	ND	23	77	3.5
**CFB**	15.2 ± 1.0^a^	7.6 ± 0.2^a^	58.7 ± 1.6^a^	17.3 ± 0.3^a^	0.5 ± 0.1	0.9 ± 0.4	24	76	3.4

ND, not detected. Different letters in the same column indicate significant differences (Tukey’s test *p* < 0.05). UFA, unsaturated fatty acid; SFA, saturated fatty acid; O/L, oleic/linoleic acid ratio.

### 3.4 Techno-functional properties

**FIGURE 4:**
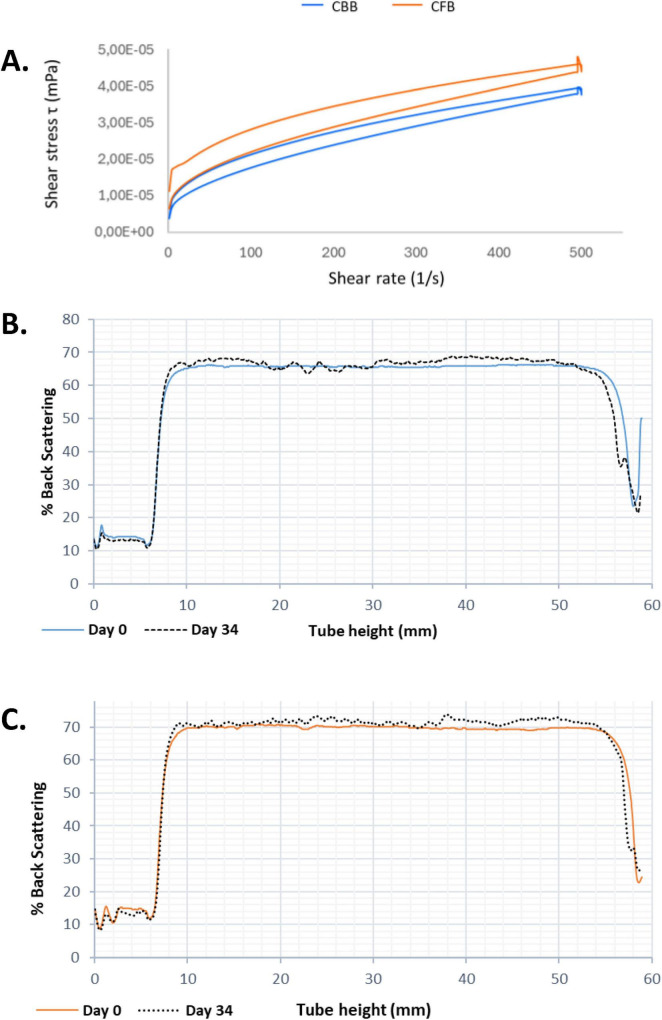
Flow curves for cashew-based beverage (CBB; blue) and cashew fermented beverage (CFB; orange) **(A)**. % Back Scattering (%BS) along the tube for CBB at day 0 and day 34 **(B).** % Back Scattering (%BS) along the tube for CFB at day 0 and day 34 **(C)**.

Figure 4A presents the flow curves corresponding to CBB and CFB. A non-Newtonian flow behavior was observed, specifically pseudoplastic and thixotropic, with the presence of a hysteresis area in both beverages which is an indicator of the degree of structural breakdown occurring during the application of shear stress. The flow curve of CFB consistently lies above that of CBB, indicating higher apparent viscosity in the fermented beverage. Apparent viscosity of CFB showed a value of 130.5 ± 0.8 mPa.s at 300 s ^−1^, significantly different compared with CBB (102.0 ± 5.0 mPa.s).

Additionally, the colloidal stability of the beverages was evaluated through laser light scattering measurements along the height of the sample cell. Two measurements per week were performed over 34 days, with samples stored at 4°C throughout the study. Figures 4B,C show the kinetics of backscattered light (%BS) as a function of sample height for CBB and CFB, respectively. Both beverages showed colloidal stability for at least 1-month of refrigeration. In the CBB, a slight increase in %BS is observed at the bottom of the tube between day 0 and day 34, indicating the onset of sedimentation after approximately 1 month of storage (Figure 4B). Regarding CFB, Figure 4C shows no increase in %BS at the bottom of the tube, nor any decrease at the top.

The CIELab lightness and color parameters of CBB and CFB are presented in Table 5. The a* and b* values for both beverages fall within a neutral or brownish tonal range (Figure 1C). Regarding the ΔE, a value of 1.17 suggests that the color difference between CBB and CFB is barely perceptible, consistent with standard visual perception ranges (Mao et al., 2024). Overall, fermentation did not produce major color changes, although significant differences (*p* < 0.05) were observed in L* and HUE parameters (Table 5).

**TABLE 5:** Color parameter values according to the CIELab scale for cashew-based beverage (CBB) and cashew fermented beverage (CFB).

Sample	L*	a*	b*	HUE	ΔE
**CBB** **CFB**	84.2 ± 0.3 ^b^	0.564 ± 0.2 ^a^	11.985 ± 0.2 ^a^	267.0 ± 1.0 ^a^	1.17 ± 0.3
	81.4 ± 0.1 ^a^	0.128 ± 0.1 ^a^	11.625 ± 0.1 ^a^	269.4 ± 0.4 ^b^	

L*(lightness), a* (red-green axis), b* (yellow-blue axis), and ΔE (color difference). Different letters in the same column indicate significant differences (Tukey’s test *p* < 0.05).

### 3.5 Anti-inflammatory properties of fermented beverage supernatants at intestinal level

**FIGURE 5:**
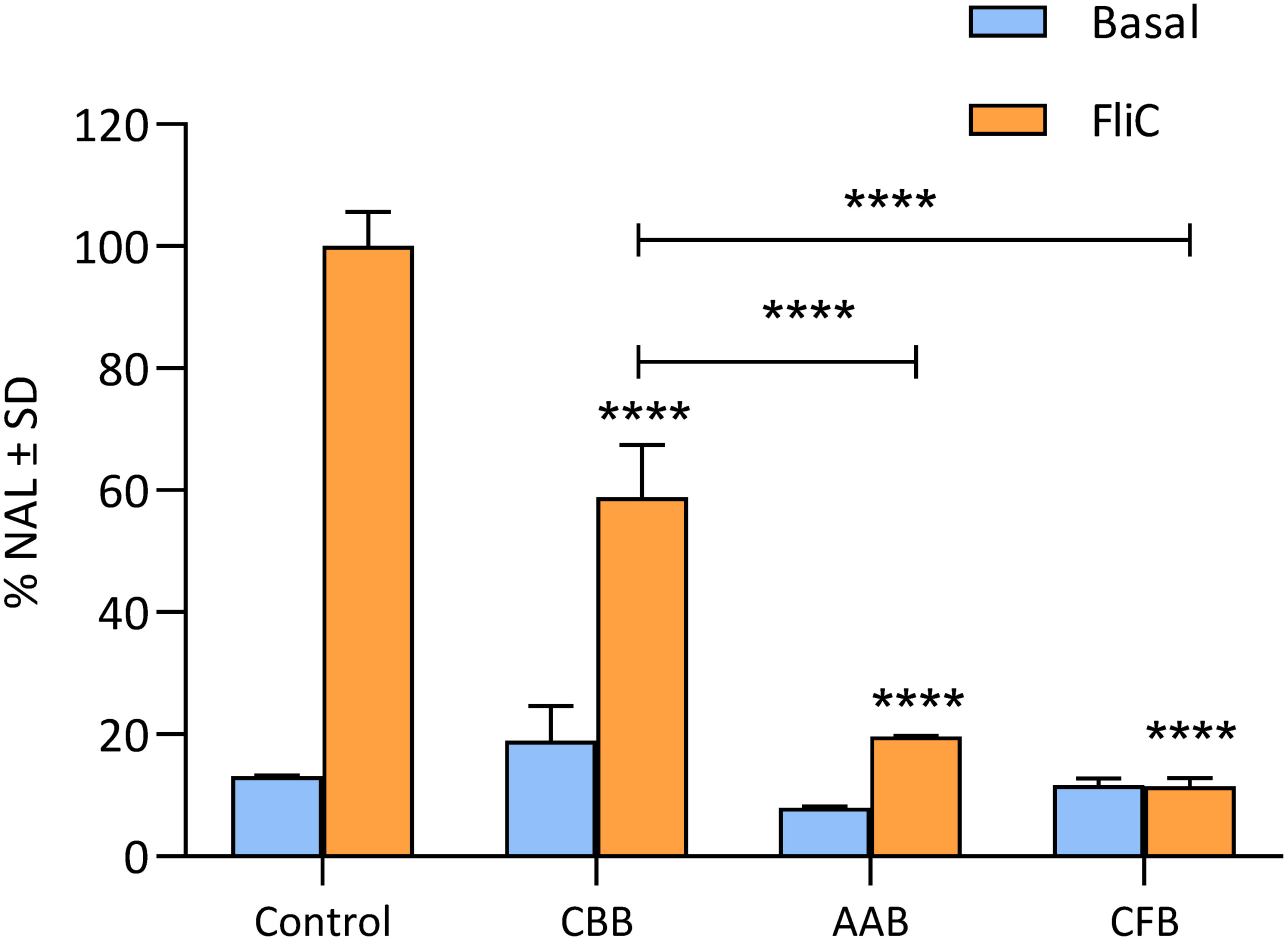
Percentage normalized average luminescence (% NAL) of Caco-2 ccl20:luc cells either untreated (CONTROL) or preincubated with neutralized cashew-based beverage (CBB), cashew fermented beverage (CFB) or artificially acidified beverage supernatants (AAB: lactic acid 50 mM). Light blue bars represent cells without stimulation (Basal condition) and orange bars correspond to cells stimulated with 1 μg/mL of Flagellin (FliC). Results are expressed as mean ± standard deviation (SD) of at least three independent experiments. Different letters indicate statistical differences among the data (*p* < 0.05; Tukey’s multiple comparison test; ****p < 0.0001).

To evaluate the effect of the bioactive compounds in the fermented cashew beverage, the anti-inflammatory properties of CFB non-microbial fraction and CBB supernatant were studied using a Caco-2 ccl20:luc reporter system. The CCL20 promoter is highly inducible by proinflammatory stimuli, like flagellin (FliC), which activates this promoter via NF-κB signaling, increasing luciferase expression. Consequently, the luciferase activity is proportional to ccl20 gene expression and reflects the activation of the innate immune response in these cells (Romanin et al., 2010). Figure 5 shows that the supernatants of CBB and CFB were able to down-regulate the innate immune response induced by FliC in intestinal epithelial cells. Cells pre-incubation with CBB led to a 50% reduction of luciferase expression, while CFB decreased it by 85%. The supernatant of the artificially acidified beverage with similar levels of lactic acid (50 mM) presented the same behavior as the fermented product indicating the role of lactic acid produced during fermentation in the anti-inflammatory properties. These results suggest that the cashew beverage contains soluble components with intestinal anti-inflammatory activity. However, fermentation with CIDCA 8327 and CIDCA 8339 produces bioactive metabolites and modifies the matrix, thereby enhancing its immunomodulatory effect.

## 4 Discussion

An ideal starter culture for non-sterile beverages must grow rapidly and acidify the product quickly to prevent spoilage by contaminating microorganisms. Additionally, it should enhance the product’s taste and aroma while improving its texture (Montemurro et al., 2021). In this study, a pasteurized cashew beverage was produced, and four LAB isolated from kefir grains were evaluated as potential starters.

While the development of fermented plant-based beverages has been a major focus in recent years, research on cashew nut fermented beverages remains limited. A cashew fermented beverage with *L. paracasei* ATCC 334 reaches 8.27 log CFU/mL of LAB after 6 h incubation (Lacerda Sousa et al., 2022). However, the authors observed no significant changes in pH during the fermentation period. In the present work, a similar result was achieved with *Lc lactis* CIDCA 8221 and *L paracasei* CIDCA 83124, which grew efficiently, showing a slight acidification of the food matrix. Huang et al. (2023) demonstrated that *Lc. lactis* mutants that were defective in sucrose utilization were not efficient at acidifying nut beverages. The authors concluded that only sucrose-utilizing strains have the potential to ferment these matrices. Consequently, it can be inferred that *Lc lactis* CIDCA 8221 and *L paracasei* CIDCA 83124 strains are unable to metabolize this sugar, which makes them unsuitable as starters for this matrix. In contrast, *L. plantarum* CIDCA 8327 and *L. paracasei* CIDCA 8339 were able to grow in the cashew-based beverage while reducing pH. Moreover, CIDCA 8339 imparted a desirable “yogurt-like” aroma that could be attributed to the production of acetaldehyde during fermentation, which is a metabolite usually responsible for the peculiar aroma of conventional yogurt (Montemurro et al., 2021).

Heating below 100°C (pasteurization) reduces vegetative bacteria, yeasts, and molds, but spore-forming bacteria can survive the process (Taormina and Hardin, 2021). In this study, a spore-forming bacillus belonging to the genus *Paenibacillus* was detected in the pasteurized cashew beverage. *Paenibacillus* is a diverse genus that includes many plant-associated species known to promote growth through phytohormone production, nutrient solubilization, and biological control mechanisms. Species of this genus can be Gram positive, Gram negative, or Gram variable. Most strains are non-pathogenic but are of concern to food manufacturers. In dairy products, *Paenibacillus* leads to defects in flavor and texture during refrigerated storage due to its cold-tolerant growth (Grady et al., 2016). *Bacillus* and *Paenibacillus* have been reported as dominate bacteria in pasteurized nut beverages. Nut milk analogs contain substrates that are supportive of microbial germination and proliferation (Taormina and Hardin, 2021). In fact, Bartula et al. (2023) demonstrated that *Paenibacillus* grew rapidly in almond and cashew beverages with a growth rate significantly higher than that evidenced in milk, negatively affecting the shelf-life of these products. In this study, *L. plantarum* CIDCA 8327 showed the capacity to completely inhibit *Paenibacillus* growth acquiring a product with prolonged shelf life.

*L. plantarum* CIDCA 8327 and *L. paracasei* CIDCA 8339 are exopolysaccharide (EPS)-producing bacteria (Hamet et al., 2015; Gangoiti et al., 2017; Bengoa et al., 2023) with promising probiotic properties previously described (Golowczyc et al., 2008; Londero et al., 2011; Zavala et al., 2016; Bengoa et al., 2018a, 2018b, 2019a, 2020b, 2020a), which make them attractive candidates to be employed as starters in novel functional foods. The use of mixed-cultures containing two or more microorganisms is gaining attention as a strategy for fermenting plant-based materials, as such combinations can lead to interactions that enhance starter growth, improve nutritional quality, and enrich the sensory profile of the final product (Tangyu et al., 2019). The mixed-culture with *L. plantarum* CIDCA 8327 and *L. paracasei* CIDCA 8339 promotes synergisms between both microorganisms, improving the fermentation process, with higher LAB counts, faster acidification, and increased organic acid levels. Similar results were reported by Shori and Al Zahrani (2022), who investigated the inclusion of three probiotic LAB strains on the production of cashew-based yogurt, alongside *Streptococcus thermophilus* and *Lactobacillus delbrueckii* subsp. *lactis*. The authors found that the presence of *L. plantarum* significantly reduced the pH compared to control yogurt without added probiotics. Cashew nut beverage fermented with CIDCA 8327 and CIDCA 8339 had final concentrations of each LAB over 10^8^ CFU/mL, surpassing the minimum level required for probiotic foods (10^6^–10^7^ CFU/mL) (Palanivelu et al., 2022). The combination of CIDCA 8327 and CIDCA 8339 favored the production of lactic acid, leading to a two-fold increase compared to CIDCA 8327 alone. Lactic acid, a key metabolite produced by LAB in fermented products, not only enhances organoleptic and antimicrobial properties but also confers health benefits by exerting an immunomodulatory activity in the gut (Garrote et al., 2015). The fermented beverage also contains butyric acid, a short-chain fatty acid known for its benefits on intestinal health. It has various positive effects, including anti-inflammatory and anti-carcinogenic properties on the colon. Additionally, butyric acid helps reinforce the intestinal epithelial barrier and modulate oxidative stress (Elfadil et al., 2023). The results obtained in this study evidence that *L. plantarum* CIDCA 8327 is responsible for the production of this acid in the cashew beverage. Previous studies have reported the biosynthesis of butyric acid by different *L. plantarum* strains, although this production seems to be strain-dependent (Botta et al., 2017; Aiello et al., 2023). Aiello et al. (2023) found that *L. plantarum* FP37, FP38, and FP48 strains produced butyric acid via lipase-mediated triglyceride hydrolysis. However, this metabolic pathway relies on tributyrin as a substrate, which is a common triglyceride found in animal fat but not present in plant-based products. On the other hand, Botta et al. (2017) conducted a genomic analysis and attributed the production of butyric acid by *L. plantarum* to the complementary activities of a medium-chain acyl-ACP thioesterase (TE) and the fatty acid synthase of type two (FASII). The authors suggested that in response to external stimuli, TE enzymes interrupt fatty acid elongation during the synthesis of membrane lipids via FASII pathway. This leads to the release of different long-chain fatty acids, including butyric acid. Furthermore, they identified glutamine as a nutritional factor that induces butyric acid production by *L. plantarum.* This finding may explain the levels of butyric acid evidenced in cashew-fermented beverages, as glutamine is the most abundant amino acid in these nuts (Gonçalves et al., 2023). In this context, and based on the results obtained, the mixed-culture with *L. plantarum* CIDCA 8327 and *L. paracasei* CIDCA 8339 strains was chosen as an appropriate starter for cashew beverage fermentation.

The superficial color of cashew beverage and the selected fermented beverage were characterized, showing a neutral or brownish tone. Jaeger et al. (2024) analyzed 18 different plant-based beverages to provide new consumer-focused insights into the sensory drivers of liking and disliking these products, representing a wide range of plant-based beverages. The authors described that color impacted sample liking differently, with a lift associated with cream color and no penalty on liking associated with light brown color.

Regarding cashew-based plant beverages composition, Reyes-Jurado et al. (2021) described a formulation containing 0.28% protein, 9.17% carbohydrates, 0.97% lipids, and 0.3% fiber. Lima Araujo Filho et al. (2023) reported a cashew-based beverage with higher solid content, including 1.83% protein, 3.97% lipids, and 5.43% carbohydrates. In this context, the beverage developed in the present work shows a significantly higher nutritional contribution in terms of macronutrients (Table 3). A similar pattern is observed in fermented cashew beverages, although few commercially available products are directly comparable to the fermented beverage produced in this study. Tangyu et al. (2019) reported that commercial plant-based beverages are neither nutritionally balanced nor comparable to animal milk. Specifically, protein content in plant-based beverages is usually low, often negligible (<0.5%), with only some soy-based milk analogs reaching higher protein levels than cow’s milk (3.7% proteins) (Jeske et al., 2017; Zannini et al., 2018). Interestingly, CBB and CFB display protein values even higher than cow’s milk, highlighting their high protein content. According to the Argentine Food Code, a product must contain at least 3% fiber to be classified as a “source of fiber.” The product developed in this study does not meet this regulatory requirement. However, a 2% fiber content still represents a meaningful nutritional contribution, particularly compared with similar commercial products, which typically contain little or no fiber. Moreover, the FDA defines a Daily Value of 28 g of dietary fiber based on a 2,000 kcal diet and classifies foods containing 1.4 g or less per serving as low in dietary fiber (Anderson et al., 2009). In this context, a 200 mL serving of CBB or CFB provides more than twice that amount.

The cashew beverages contained high lipid levels (8–9%), mainly naturally occurring healthy fatty acids from cashew nuts, which contribute to their nutritional value. The fatty acid profile was consistent with that reported by Rico et al. (2016). The authors determined the nutrient composition of raw fresh cashew kernels from the major world-producing areas, identifying oleic acid as the most abundant fatty acid, with a contribution of 60.7% to the total fat, followed by linoleic 17.77%, palmitic 10.2%, and stearic 8.93% acids. The results indicate that the beverage fatty acid profile matches that of cashew nuts. Although the lipid content was high, more than 75% of the fatty acids were unsaturated (Table 4). Liu et al. (2023) reported that saturated fatty acids (SFA) are associated with adverse health effects and are considered risk factors for obesity and cardiovascular disease. In contrast, unsaturated fatty acids (UFA) have been linked to protective effects against several diseases (Rico et al., 2016). Therefore, CBB and CFB represent promising candidates for nutritional applications.

A major challenge in developing fermented plant-based beverages is achieving the viscosity and mouthfeel expected of yogurt-like products. Although gums and hydrocolloids can be effective, consumer demand for clean-label foods discourages the use of additives. In this context, EPS-producing LAB represent a promising starter alternative. Apparent viscosity of CFB in this study falls within the range reported for fermented plant-based beverages in the literature. Pérez Martínez et al. (2010) reported that drinkable yogurts or fermented milks typically have apparent viscosity values ranging from 60 to 250 mPa.s. Authors inoculated a hazelnut beverage with *St. thermophilus* BS5 *and L. acidophilus BL228*, reaching an apparent viscosity of 110 mPa.s after fermentation at 37°C. The greater viscosity of CFB compared with CBB resulted from fermentation. During this process, bacteria convert sugars into organic acids, lowering pH and inducing protein coagulation, which leads to a more viscous structure (Robinson, 2005). Moreover, *L. paracasei* CIDCA 8339 and *L. plantarum* CIDCA 8327 are capable of producing EPS in milk and culture media (Gangoiti et al., 2017; Bengoa et al., 2018a,^2023^). EPS produced by LAB play a key role in the rheology, texture, and mouthfeel of fermented products and are widely used as natural thickening agents (Abarquero et al., 2022). Creamy texture in yogurts, one of the most desirable quality attributes for consumers, is often associated with EPS production by the fermenting strains (Torino et al., 2015; Abarquero et al., 2022). In this context, the higher viscosity observed in CFB may be partly attributed to EPS production by the starter during fermentation. *In situ* EPS production may also improve product stability and water-holding capacity, thereby reducing phase separation during refrigeration (Montemurro et al., 2021). *In situ* EPS production may also improve product stability and water-holding capacity, thereby reducing phase separation during refrigeration. Bernat et al. (2015) evaluated the colloidal stability of almond-based beverages stored at 4°C for 28 days, observing phase separation and flocculation evidence from the first day. Stable beverages were only obtained when heat treatments and homogenization were applied to improve stability. Similarly, Manassero et al. (2020) studied amaranth-based beverages and reported that those without stabilizers presented a visible phase separation after 7 days, reaching up to 19% separation after 30 days. In contrast, cashew beverage maintained colloidal stability for at least 1 month, evidencing only a slight onset of sedimentation after storage. Moreover, fermentation did not affect beverage stability. This behavior may be attributed to the viscosity rise induced by fermentation, previously described. Higher viscosity increases resistance to particle movement, reducing sedimentation rate and minimizing the tendency of oil droplets to flocculate in the fermented beverage.

The consumption of functional foods with probiotics could help to alleviate gut inflammation, either by microbiota modulation or by the anti-inflammatory properties exerted by the microorganism and its metabolites (Cristofori et al., 2021; Dahiya and Nigam, 2022). The cashew-based beverage developed in this study showed anti-inflammatory properties at the intestinal level. In agreement, Siracusa et al. (2020) and Fusco et al. (2020) demonstrated that oral administration of cashew nuts exhibits anti-inflammatory effects in murine models, an effect mediated by the inhibition of the NF-κB signaling pathway. Among the wide variety of bioactive compounds present in nuts, phytochemicals (phytosterols and polyphenols) and fatty acids are the main candidates proposed as responsible for their anti-inflammatory effects. Antioxidants such as γ-tocopherol suppress inflammation through the inhibition of NF-κB (Rajaram et al., 2023). Furthermore, oleic acid is considered an anti-inflammatory compound since it significantly reduces the production of pro-inflammatory cytokines in DSS-challenged Caco-2 cells and LPS-stimulated macrophages (Charlet et al., 2022). In this context, the high proportion of oleic acid in CBB may contribute to its immunomodulatory effect. Fermentation of CBB with the mixed starter enhanced this effect. The results suggest that organic acids, mainly lactic acid, produced by *L. plantarum* CIDCA 8327 and *L. paracasei* CIDCA 8339 during fermentation are key metabolites underlying the anti-inflammatory effect observed in CFB. Previous works have reported that lactic acid modulates key players of innate response, such as myeloid and epithelial cells (Garrote et al., 2015). Iraporda et al. (2014) proved that the organic acids present in kefir were responsible for the immunomodulatory activity of the non-microbial fraction of this fermented milk at the intestinal level. Moreover, lactate reduced intestinal inflammation and epithelial damage in an *in vivo* TNBS-induced colitis model (Iraporda et al., 2016). In addition, minor organic acids in CFB, such as butyric and acetic acids, have shown intestinal anti-inflammatory activity (Iraporda et al., 2015) and may contribute to this effect. As shown in Section 3.2, use of the mixed culture *L. plantarum* CIDCA 8327 and *L. paracasei* CIDCA 8339 increased organic acid production in the fermented beverage, thereby enhancing the health-promoting properties of the final product.

## 5 Conclusion

The results of the present study propose the probiotic bacteria *L. plantarum* CIDCA 8327 and *L. paracasei* CIDCA 8339 isolated from kefir grains as outstanding candidates for developing a cashew-based fermented beverage. The fermented product represents a non-dairy alternative source of beneficial probiotic microorganisms with favorable nutritional characteristics, particularly its high contents of protein, fiber, and healthy fatty acids. Fermentation with this mixed-culture enhances shelf life by inhibiting the development of potential spoilage microbes, while also improving the product’s techno-functional properties and beneficial health effects through the production of anti-inflammatory compounds. In conclusion, cashew-based beverages provide a suitable matrix for the growth of kefir-isolated LAB and represent a promising platform for developing functional plant-based foods that may enhance consumers’ gastrointestinal health.

## Data Availability

The gene sequence was deposited in Genbank (NCBI, Accession number PX987639).

## Footnotes

1
https://blast.ncbi.nlm.nih.gov/Blast.cgi
