# Supplementary material for: Application of kefir-isolated Lactiplantibacillus plantarum and Lacticaseibacillus paracasei to obtain a cashew-based fermented beverage with enhanced anti-inflammatory properties
Source: Front Microbiol. 2026 Mar 10;17:1763414. doi: 10.3389/fmicb.2026.1763414 (PMC13008870; doi:10.3389/fmicb.2026.1763414)
Supplement: Supplementary file 1 [file Data_Sheet_1.pdf]

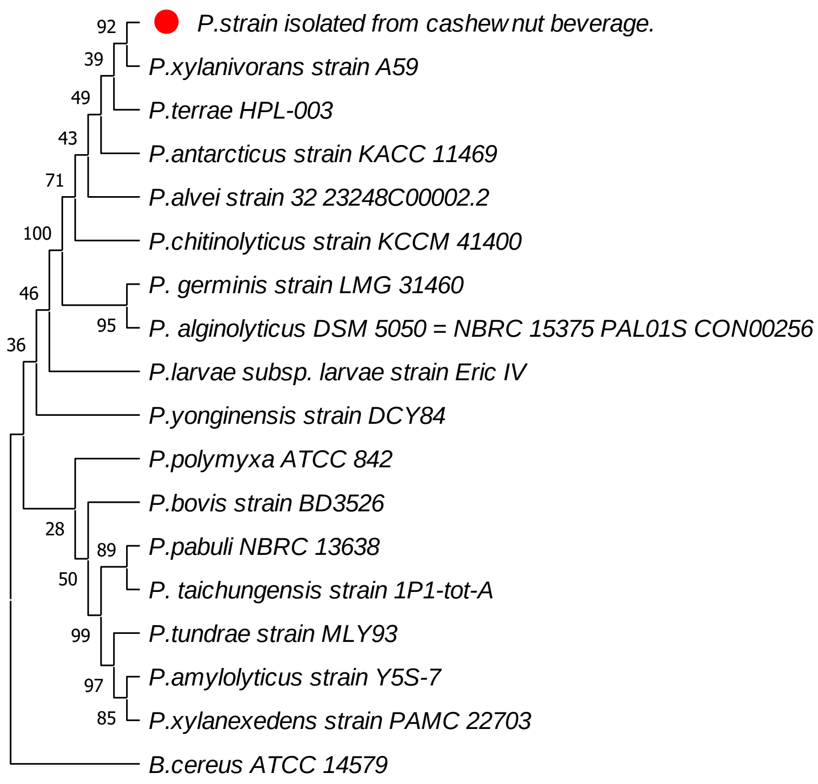

Figure S1. Phylogenetic tree based on 16S rRNA gene sequences constructed in MEGA X (v11) using the Neighbor-Joining method and Kimura two-parameter distances. Bootstrap support was estimated with 1,000 replicates, and a bootstrap consensus tree is shown. *Bacillus cereus* was included as the outgroup.
